# Estimating the purebred–crossbred genetic correlation for uniformity of eggshell color in laying hens

**DOI:** 10.1186/s12711-016-0212-2

**Published:** 2016-05-05

**Authors:** Han A. Mulder, Jeroen Visscher, Julien Fablet

**Affiliations:** Animal Breeding and Genomics Centre, Wageningen University & Research, PO Box 338, 6700 AH Wageningen, The Netherlands; Institut de Sélection Animale B.V., Hendrix Genetics, PO Box 114, 5830 AC Boxmeer, The Netherlands; Institut de Sélection Animale S.A.S., Hendrix Genetics, 22440 Ploufragan, France

## Abstract

**Background:**

Uniformity of eggs is an important aspect for retailers because consumers prefer homogeneous products. One of these characteristics is the color of the eggshell, especially for brown eggs. Existence of a genetic component in environmental variance would enable selection for uniformity of eggshell color. Therefore, the objective of this study was to quantify the genetic variance in environmental variance of eggshell color in purebred and crossbred laying hens, to estimate the genetic correlation between environmental variance of eggshell color in purebred and crossbred laying hens and to estimate genetic correlations between environmental variance at different times of the laying period.

**Methods:**

We analyzed 167,651 and 79,345 eggshell color records of purebred and crossbred laying hens, respectively. The purebred and crossbred laying hens originated mostly from the same sires. Since eggshell color records of crossbred laying hens were collected per cage, these records could be related only to cage and sire family. A double hierarchical generalized linear sire model was used to estimate the genetic variance of the mean of eggshell color and its environmental variance. Approximate standard errors for heritability and the genetic coefficient of variation for environmental variance were derived.

**Results:**

The genetic variance in environmental variance at the log scale was equal to 0.077 and 0.067, for purebred and crossbred laying hens, respectively. The genetic coefficient of variation for environmental variance was equal to 0.28 and 0.26, for purebred and crossbred laying hens, respectively. A genetic correlation of 0.70 was found between purebred and crossbred environmental variance of eggshell color, which indicates that there is some reranking of sires for environmental variance of eggshell color in purebred and crossbred laying hens. Genetic correlations between environmental variance of eggshell color in different laying periods were generally higher than 0.85, except between early laying and mid or late laying periods.

**Conclusions:**

Our results indicate that genetic selection can be efficient to improve uniformity of eggshell color in purebreds and crossbreds, ideally by applying combined crossbred and purebred selection. This methodology can be used to estimate genetic correlations between purebred and crossbred lines for uniformity of other traits and species.

**Electronic supplementary material:**

The online version of this article (doi:10.1186/s12711-016-0212-2) contains supplementary material, which is available to authorized users.

## Background

Animal products require a certain level of homogeneity. In some cases, homogeneity or uniformity has benefits for product processing, e.g. meat [1], and retailers and their customers usually prefer uniform meat cuts. Eggs need to be uniform with respect to size, weight, and eggshell color in the case of some brown egg markets. Heritabilities for eggshell color are moderate to high, 0.4 to 0.7 [2, 3]; it should be noted that these heritability estimates were based on averages of a number of eggs collected per hen. Such heritability estimates along with the large genetic variance show that eggshell color can be easily changed by selection in the direction of dark brown or light brown eggs. However, selection on eggshell color does not necessarily make the eggs uniform and to date, there is no evidence that selection for more uniform brown eggs is possible.

Selection for more uniform brown eggs requires the presence of genetic variation in the uniformity of this trait. For several other traits, there is empirical evidence for the existence of genetic variance in environmental variance ($V_{\text{E}}$). Typically, the genetic standard deviation expressed relative to the mean, i.e. the genetic coefficient of variation ($GCV_{Ve}$), is ~0.3 [4], which indicates that if the selection response in $V_{\text{E}}$ is equal to one genetic standard deviation (e.g. a selection intensity of 2.0 and an accuracy of 0.5), then $V_{\text{E}}$ would change by 30 %. Heritabilities of $V_{\text{E}}$ that are expressed at the individual phenotypic record level are generally low and range from 0.01 to 0.05, while heritabilities of 0.1 were found for within-litter variation of birth weight of piglets [5, 6] or standard deviation of egg weight [7]. In other words, high accuracies of selection could be obtained, at least for selection on the sires. Eggshell color is measured several times during a laying period, which provides the opportunity to study genetic variation in $V_{\text{E}}$ of eggshell color at different times of the egg laying period. Genetic variation in $V_{\text{E}}$ may differ between laying periods and genetic correlations between $V_{\text{E}}$ in different laying periods may differ from 1.

In pigs and poultry, the breeding goals are directed towards increasing performance at the crossbred level, whereas selection is performed at the purebred level. For example, in laying hens recurrent test selection schemes are used to select simultaneously on purebred and crossbred performance. Wei and Van der Werf [8] and Besbes and Gibson [9] found genetic correlations between 0.56 and 0.99 and between 0.8 and 0.94, respectively, for egg laying traits in purebred and crossbred laying hens. In pigs, the genetic correlations between purebred and crossbred performances range for most traits from 0.7 to 0.9 [10–12]. The genetic correlation between purebred and crossbred performances is the key parameter for determining the need for crossbred information in breeding schemes [13, 14]. The genetic correlation between $V_{\text{E}}$ of eggshell color in purebred and crossbred laying hens is, however, unknown.

Therefore, the objectives of this study were to estimate the genetic variance in $V_{\text{E}}$ of eggshell color in purebred and crossbred laying hens, to estimate the genetic correlation between $V_{\text{E}}$ in purebred and crossbred laying hens and to estimate genetic correlations between $V_{\text{E}}$ in different laying periods.

## Methods

### Data

Eggshell color was measured on individual eggs during four periods on purebred hens (period 1: 25 to 35 weeks, period 2: 36 to 55 weeks, period 3: 56 to 75 weeks and period 4: 76 to 95 weeks of age) and during three periods in crossbred hens (30 to 45, 50 to 65 and 70 to 85 weeks of age). Purebred hens were individually housed, whereas crossbred hens were housed as paternal half-sibs in group cages that contained between four and 17 hens. Eggshell color was measured with a reflectometer (Minolta) using three parameters: L* measures lightness (0 is black; 100 white), a* measures hue as a function of the red–green scale (<0 is green; >0 is red) and b* measures hue as a function of the blue–yellow scale (<0 is blue, >0 is yellow) [15]. The three measures were combined into an eggshell color index as L*–a*–b* and multiplied by 10. Data were collected on purebred hens between 2006 and 2013 and on crossbred hens between 2009 and 2013. The raw data contained 221,467 records for purebreds and 96,106 for crossbreds. For purebreds, we limited the data to have at least five records per hen in order to estimate permanent environmental effects and to have at least 40 daughters per sire resulting in 167,651 records for analysis. For crossbreds, at least 40 records per sire were required resulting in 79,345 records after editing. Crossbred and purebred hens had a sire from the same line, whereas their dams originated from four different female lines. In total, 279 sires had purebred daughters and 880 sires had crossbred daughters, while 71 sires had both purebred and crossbred daughters. The sire pedigree was traced back 5 generations and contained 2491 animals. The summary statistics of the data are in Table 1. Data showed some skewness and kurtosis.

**Table 1:** Summary statistics of purebred and crossbred eggshell color data after editing

	Purebred	Crossbred
Number of records	167,651	79,345
Average	206.40	189.70
SD	90.60	85.55
Median	198.00	181.00
Minimum	−60.00	−61.00
Maximum	606.00	782.00
Skewness	0.49	0.73
Kurtosis	3.14	4.24

### Estimation of the genetic correlations between purebred and crossbred performance using DHGLM

The main aim was to estimate the genetic correlation between $V_{\text{E}}$ eggshell color in purebreds and crossbreds. Due to differences in housing, the definition of $V_{\text{E}}$ differed. In purebreds, $V_{\text{E}}$ was the within-individual variance of eggshell color because repeated observations per hen were available. In crossbreds, $V_{\text{E}}$ contained both within-individual variance and between-hen variance. The between-hen variance was partly due to genetic differences because only the sire was known and to non-genetic effects such as permanent environmental effects. The difference in definition between $V_{\text{E}}$ in purebreds and crossbreds may affect the genetic correlation between purebreds and crossbreds. This was further investigated by performing a simulation based on purebred data, see the section ‘Effect of different definitions of environmental variance’.

The genetic analysis of $V_{\text{E}}$ was based on the double hierarchical generalized linear model (DHGLM) [16, 17]. Here, we extended the model to estimate simultaneously genetic variance in $V_{\text{E}}$ in purebred and crossbred laying hens. Because the DHGLM modeled the level and the variance of a trait, the analysis became a 4 × 4 analysis. A sire model was used because the links between purebreds and crossbreds depended on the sires and, in crossbreds, the eggs were collected at the cage level and the hens were housed as paternal half-sibs. For purebreds, we used random permanent environmental effects to account for genetic (dam genetic effect, Mendelian sampling effect, dominance and epistasis) and non-genetic permanent environmental effects. For crossbreds, we used random cage effects to account for potential cage effects. Because for crossbreds, we did not know which hen produced which egg, permanent environmental effects could not be fitted. Therefore, the residual variance of crossbreds contained three-quarters of the additive genetic variance $V_{\text{A}}$ (residual variance = $V_{\text{E}} + 0.75V_{\text{A}}$), whereas, in purebreds, this was absorbed by the permanent environmental effect (residual variance = $V_{\text{E}}$ = within-individual variance). In Mulder et al. [18], a sire model adjustment was shown to account for the fact that the residual variance contained three-quarters of the genetic variance. Therefore, we applied the sire model adjustment of Mulder et al. [18] for crossbreds. The bivariate DHGLM becomes:

1 $$\begin{aligned} \left[ {\begin{array}{*{20}c} {{\mathbf{y}}_{{\mathbf{p}}} } \\ {{\mathbf{y}}_{{\mathbf{c}}} } \\ {\begin{array}{*{20}c} {{\mathbf{y}}_{{{\mathbf{v}}_{{\mathbf{p}}} }} } \\ {{\mathbf{y}}_{{{\mathbf{v}}_{{\mathbf{c}}} }} } \\ \end{array} } \\ \end{array} } \right] & = \left[ {\begin{array}{*{20}c} {\begin{array}{*{20}c} {{\mathbf{X}}_{{\mathbf{p}}} } & {\mathbf{0}} \\ {\mathbf{0}} & {{\mathbf{X}}_{{\mathbf{c}}} } \\ \end{array} } & {\begin{array}{*{20}c} {\mathbf{0}} & {\mathbf{0}} \\ {\mathbf{0}} & {\mathbf{0}} \\ \end{array} } \\ {\begin{array}{*{20}c} {\mathbf{0}} & {\mathbf{0}} \\ {\mathbf{0}} & {\mathbf{0}} \\ \end{array} } & {\begin{array}{*{20}c} {{\mathbf{X}}_{{{\mathbf{v}}_{{\mathbf{p}}} }} } & {\mathbf{0}} \\ {\mathbf{0}} & {{\mathbf{X}}_{{{\mathbf{v}}_{{\mathbf{c}}} }} } \\ \end{array} } \\ \end{array} } \right]\left[ {\begin{array}{*{20}c} {{\mathbf{b}}_{{\mathbf{p}}} } \\ {{\mathbf{b}}_{{\mathbf{c}}} } \\ {\begin{array}{*{20}c} {{\mathbf{b}}_{{{\mathbf{v}}_{{\mathbf{p}}} }} } \\ {{\mathbf{b}}_{{{\mathbf{v}}_{{\mathbf{c}}} }} } \\ \end{array} } \\ \end{array} } \right] \\ & \quad + \left[ {\begin{array}{*{20}c} {\begin{array}{*{20}c} {{\mathbf{Z}}_{{\mathbf{p}}} } & {\mathbf{0}} \\ {\mathbf{0}} & {{\mathbf{Z}}_{{\mathbf{c}}} } \\ \end{array} } & {\begin{array}{*{20}c} {\mathbf{0}} & {\mathbf{0}} \\ {\mathbf{0}} & {\mathbf{0}} \\ \end{array} } \\ {\begin{array}{*{20}c} {\mathbf{0}} & {\mathbf{0}} \\ {\mathbf{0}} & {\mathbf{0}} \\ \end{array} } & {\begin{array}{*{20}c} {{\mathbf{Z}}_{{{\mathbf{v}}_{{\mathbf{p}}} }} } & {\mathbf{0}} \\ {\mathbf{0}} & {{\mathbf{Z}}_{{{\mathbf{v}}_{{\mathbf{c}}} }} } \\ \end{array} } \\ \end{array} } \right]\left[ {\begin{array}{*{20}c} {{\mathbf{s}}_{{\mathbf{p}}} } \\ {{\mathbf{s}}_{{\mathbf{c}}} } \\ {\begin{array}{*{20}c} {{\mathbf{s}}_{{{\mathbf{v}}_{{\mathbf{p}}} }} } \\ {{\mathbf{s}}_{{{\mathbf{v}}_{{\mathbf{c}}} }} } \\ \end{array} } \\ \end{array} } \right] \\ & \quad + \left[ {\begin{array}{*{20}c} {\begin{array}{*{20}c} {{\mathbf{V}}_{{\mathbf{p}}} } & {\mathbf{0}} \\ {\mathbf{0}} & {{\mathbf{U}}_{{\mathbf{c}}} } \\ \end{array} } & {\begin{array}{*{20}c} {\mathbf{0}} & {\mathbf{0}} \\ {\mathbf{0}} & {\mathbf{0}} \\ \end{array} } \\ {\begin{array}{*{20}c} {\mathbf{0}} & {\mathbf{0}} \\ {\mathbf{0}} & {\mathbf{0}} \\ \end{array} } & {\begin{array}{*{20}c} {{\mathbf{V}}_{{{\mathbf{v}}_{{\mathbf{p}}} }} } & {\mathbf{0}} \\ {\mathbf{0}} & {{\mathbf{U}}_{{{\mathbf{v}}_{{\mathbf{c}}} }} } \\ \end{array} } \\ \end{array} } \right]\left[ {\begin{array}{*{20}c} {\begin{array}{*{20}c} {{\mathbf{pe}}_{{\mathbf{p}}} } \\ {{\mathbf{cg}}_{{\mathbf{c}}} } \\ {{\mathbf{pe}}_{{{\mathbf{v}}_{{\mathbf{p}}} }} } \\ \end{array} } \\ {{\mathbf{cg}}_{{{\mathbf{v}}_{{\mathbf{c}}} }} } \\ \end{array} } \right] + \left[ {\begin{array}{*{20}c} {{\mathbf{e}}_{{\mathbf{p}}} } \\ {{\mathbf{e}}_{{\mathbf{c}}} } \\ {\begin{array}{*{20}c} {{\mathbf{e}}_{{{\mathbf{v}}_{{\mathbf{p}}} }} } \\ {{\mathbf{e}}_{{{\mathbf{v}}_{{\mathbf{c}}} }} } \\ \end{array} } \\ \end{array} } \right], \\ \end{aligned}$$

where ${\mathbf{y}}_{{\mathbf{p}}}$ (${\mathbf{y}}_{{\mathbf{c}}}$) is the vector with eggshell color observations of purebred hens (crossbred hens), ${\mathbf{y}}_{{{\mathbf{v}}_{{\mathbf{p}}} }}$ (${\mathbf{y}}_{{{\mathbf{v}}_{{\mathbf{c}}} }}$) is the response variable for the variance model for purebred hens (crossbred hens), ${\mathbf{X}}$ and ${\mathbf{Z}}$ are the design matrices that link observations to fixed effects and sire effects, respectively, ${\mathbf{V}}$ is the design matrix that links the purebred observations to permanent environmental effects, ${\mathbf{U}}$ is the design matrix that links crossbred observations to cage effects, ${\mathbf{b}}$ is a vector of fixed effects, ${\mathbf{s}}_{{\mathbf{p}}}$, ${\mathbf{s}}_{{\mathbf{c}}}$, ${\mathbf{s}}_{{{\mathbf{v}}_{{\mathbf{p}}} }}$, and ${\mathbf{s}}_{{{\mathbf{v}}_{{\mathbf{c}}} }}$ are vectors of random sire genetic effects for purebred and crossbred eggshell color and its variance, ${\mathbf{pe}}_{{\mathbf{p}}}$ and ${\mathbf{pe}}_{{{\mathbf{v}}_{{\mathbf{p}}} }}$ are vectors of random permanent environmental effects for eggshell color and its variance in purebreds, ${\mathbf{cg}}_{{\mathbf{c}}}$ and ${\mathbf{cg}}_{{{\mathbf{v}}_{{\mathbf{c}}} }}$ are vectors of random cage effects for eggshell color and its variance in crossbreds and ${\mathbf{e}}_{{\mathbf{p}}}$, ${\mathbf{e}}_{{\mathbf{c}}}$, ${\mathbf{e}}_{{{\mathbf{v}}_{{\mathbf{p}}} }}$, and ${\mathbf{e}}_{{{\mathbf{v}}_{{\mathbf{c}}} }}$ are vectors of random residuals. For purebreds, the fixed effects were hatch week and laying date. For crossbreds, the fixed effects were line and tier effect nested within the recurrent test. The response variables ${\mathbf{y}}_{{{\mathbf{v}}_{{\mathbf{p}}} }}$ were linearized working variables following Felleki et al. [17]. In Rönnegård et al. [16], a Gamma link function was used for the variance model $\log \left( {\phi_{i} } \right) = { \log }(e_{i}^{2} /\left( {1 - h_{i} } \right))$, where $e_{i}^{2}$ is the squared residual from $y_{i}$ and $h_{i}$ is the leverage, the diagonal element of the hat matrix of ${\mathbf{y}}_{{\mathbf{p}}}$ and ${\mathbf{y}}_{{\mathbf{c}}}$ corresponding to observation $i$ [19]. Felleki et al. [17] showed that instead of using a log link function, ${ \log }\left( {e_{i}^{2} /\left( {1 - h_{i} } \right)} \right)$ can be linearized using the Taylor expansion of the first order by calculating the response variables ${\mathbf{y}}_{{{\mathbf{v}}_{{\mathbf{p}}} }}$ for observation $i$ as $y_{{v_{p,i} }} = \log \left( {\widehat{{\sigma_{{e_{i} }}^{2} }}} \right) + \frac{{\left( {e_{i}^{2} /\left( {1 - h_{i} } \right)} \right) - \widehat{{\sigma_{{e_{i} }}^{2} }}}}{{\widehat{{\sigma_{{e_{i} }}^{2} }}}}$, where $\sigma_{{e_{i} }}^{2}$ is the predicted residual variance for observation $i$. Note that $y_{{v_{i} }}$ is the linearized working variable for $\log \left( {\phi_{i} } \right)$ in the notation of Rönnegård et al. [16]. The response variables ${\mathbf{y}}_{{{\mathbf{v}}_{{\mathbf{c}}} }}$ are calculated similarly as ${\mathbf{y}}_{{{\mathbf{v}}_{{\mathbf{p}}} }}$, but with the sire model adjustment following Mulder et al. [18]. The response variables ${\mathbf{y}}_{{{\mathbf{v}}_{{\mathbf{c}}} }}$ were calculated for observation $i$ as:

$$y_{{v_{c,i} }} = \log \left( {\widehat{{\sigma_{{e_{i} }}^{2} }}} \right) + \frac{{\left( {\left( {e_{i}^{2} /\left( {1 - h_{i} } \right)} \right)*\left( {\widehat{{\sigma_{{e_{s} }}^{2} }}/\widehat{{\sigma_{{e_{a} }}^{2} }}} \right)} \right) - \widehat{{\sigma_{{e_{i} }}^{2} }}}}{{\widehat{{\sigma_{{e_{i} }}^{2} }}}},$$

where $\widehat{{\sigma_{{e_{s} }}^{2} }}$ is the residual variance of the sire model assuming homogeneous residual variance and $\widehat{{\sigma_{{e_{a} }}^{2} }} = \widehat{{\sigma_{{e_{s} }}^{2} }} - \frac{3}{4}\widehat{{\sigma_{{a_{c} }}^{2} }}$, the residual variance of an animal model. To achieve better convergence, $\widehat{{\sigma_{{e_{s} }}^{2} }}/\widehat{{\sigma_{{e_{a} }}^{2} }}$ was not updated, in which the algorithm differed from [18]. The sire genetic effects were assumed multivariate normally distributed:

$$\left[ {\begin{array}{*{20}c} {{\mathbf{s}}_{{\mathbf{p}}} } \\ {{\mathbf{s}}_{{\mathbf{c}}} } \\ {\begin{array}{*{20}c} {{\mathbf{s}}_{{{\mathbf{v}}_{{\mathbf{p}}} }} } \\ {{\mathbf{s}}_{{{\mathbf{v}}_{{\mathbf{c}}} }} } \\ \end{array} } \\ \end{array} } \right]\sim{\text{N}}\left( {\left[ {\begin{array}{*{20}c} {\mathbf{0}} \\ {\mathbf{0}} \\ {\begin{array}{*{20}c} {\mathbf{0}} \\ {\mathbf{0}} \\ \end{array} } \\ \end{array} } \right],\frac{1}{4}{\mathbf{G}} \otimes {\mathbf{A}}} \right),$$

where $${\mathbf{G}} = \left[ {\begin{array}{cccc} {\begin{array}{cc} {\sigma_{{a_{p} }}^{2} } & \quad{cov_{{a_{pc} }} } \\ &\quad {\sigma_{{a_{c} }}^{2} } \\ \end{array} } &\quad {\begin{array}{*{20}c} {cov_{{a_{p,vp} }} } &\quad {cov_{{a_{p,vc} }} } \\ {cov_{{a_{c,vp} }} } &\quad {cov_{{a_{c,vc} }} } \\ \end{array} } \\ {\begin{array}{*{20}c} &\quad \\ {symmetric} &\quad \\ \end{array} } &\quad {\begin{array}{*{20}c} {\sigma_{{a_{vp} }}^{2} } &\quad {cov_{{a_{v,pc} }} } \\ &\quad {\sigma_{{a_{vc} }}^{2} } \\ \end{array} } \\ \end{array} } \right]$$ with all the genetic (co)variances for the corresponding sire genetic effects. The permanent environmental effects and cage effects were assumed bivariate normally distributed:

$$\left[ {\begin{array}{*{20}c} {{\mathbf{pe}}_{{\mathbf{p}}} } \\ {{\mathbf{pe}}_{{{\mathbf{v}}_{{\mathbf{p}}} }} } \\ \end{array} } \right]\sim{\text{N}}\left( {\left[ {\begin{array}{*{20}c} {\mathbf{0}} \\ {\mathbf{0}} \\ \end{array} } \right], \left[ {\begin{array}{*{20}c} {\sigma_{{pe_{p} }}^{2} } & {cov_{{pe_{p,vp} }} } \\ {cov_{{pe_{p,vp} }} } & {\sigma_{{pe_{{v_{p} }} }}^{2} } \\ \end{array} } \right] \otimes {\mathbf{I}}} \right)$$

and

$$\left[ {\begin{array}{*{20}c} {{\mathbf{cg}}_{{\mathbf{c}}} } \\ {{\mathbf{cg}}_{{{\mathbf{v}}_{{\mathbf{c}}} }} } \\ \end{array} } \right]\sim{\text{N}}\left( {\left[ {\begin{array}{*{20}c} {\mathbf{0}} \\ {\mathbf{0}} \\ \end{array} } \right], \left[ {\begin{array}{*{20}c} {\sigma_{{cg_{c} }}^{2} } & {cov_{{cg_{c,vc} }} } \\ {cov_{{cg_{c,vc} }} } & {\sigma_{{cg_{{v_{c} }} }}^{2} } \\ \end{array} } \right] \otimes {\mathbf{I}}} \right),$$

with the corresponding permanent environmental (co)variances and cage (co)variances. The residuals ${\mathbf{e}}_{{\mathbf{p}}}$, ${\mathbf{e}}_{{\mathbf{c}}}$, ${\mathbf{e}}_{{{\mathbf{v}}_{{\mathbf{p}}} }}$, and ${\mathbf{e}}_{{{\mathbf{v}}_{{\mathbf{c}}} }}$ were multivariate normally distributed:

$$\left[ {\begin{array}{*{20}c} {{\mathbf{e}}_{{\mathbf{p}}} } \\ {{\mathbf{e}}_{{\mathbf{c}}} } \\ {\begin{array}{*{20}c} {{\mathbf{e}}_{{{\mathbf{v}}_{{\mathbf{p}}} }} } \\ {{\mathbf{e}}_{{{\mathbf{v}}_{{\mathbf{c}}} }} } \\ \end{array} } \\ \end{array} } \right]\sim{\text{N}}\left( {\left[ {\begin{array}{*{20}c} {\mathbf{0}} \\ {\mathbf{0}} \\ {\begin{array}{*{20}c} {\mathbf{0}} \\ {\mathbf{0}} \\ \end{array} } \\ \end{array} } \right],\left[ {\begin{array}{*{20}c} {\begin{array}{*{20}c} {{\mathbf{W}}_{{\mathbf{p}}}^{{ - {\mathbf{1}}}} \sigma_{{\varepsilon_{p} }}^{2} } & {\mathbf{0}} \\ {\mathbf{0}} & {{\mathbf{W}}_{{\mathbf{c}}}^{{ - {\mathbf{1}}}} \sigma_{{\varepsilon_{c} }}^{2} } \\ \end{array} } & {\begin{array}{*{20}c} {\mathbf{0}} & {\mathbf{0}} \\ {\mathbf{0}} & {\mathbf{0}} \\ \end{array} } \\ {\begin{array}{*{20}c} {\mathbf{0}} & {\mathbf{0}} \\ {\mathbf{0}} & {\mathbf{0}} \\ \end{array} } & {\begin{array}{*{20}c} {{\mathbf{W}}_{{{\mathbf{v}},{\mathbf{p}}}}^{{ - {\mathbf{1}}}} \sigma_{{\varepsilon_{{v_{p} }} }}^{2} } & {\mathbf{0}} \\ {\mathbf{0}} & {{\mathbf{W}}_{{{\mathbf{v}},{\mathbf{c}}}}^{{ - {\mathbf{1}}}} \sigma_{{\varepsilon_{{v_{c} }} }}^{2} } \\ \end{array} } \\ \end{array} } \right]} \right),$$

where $${\mathbf{W}}_{{\mathbf{p}}} = {\text{diag}} \left[ {{ \exp }\left( {\widehat{{{\mathbf{y}}_{{{\mathbf{v}}_{{\mathbf{p}}} }} }}} \right)} \right]^{ - 1} ,$$

$${\mathbf{W}}_{{\mathbf{c}}} = {\text{diag}}\left[ {\widehat{{\sigma_{{e_{a} }}^{2} }}\left( {\exp \left( {\widehat{{{\mathbf{y}}_{{{\mathbf{v}}_{{\mathbf{c}}} }} }}} \right)/\overline{{\exp \left( {\widehat{{{\mathbf{y}}_{{{\mathbf{v}}_{{\mathbf{c}}} }} }}} \right)}} } \right) + \frac{3}{4}\widehat{{\sigma_{{a_{c} }}^{2} }}} \right]^{ - 1} ,$$

$${\mathbf{W}}_{{{\mathbf{v}},{\mathbf{p}}}} = {\text{diag}}\left[ {\frac{1}{2}\left( {1 - {{\mathbf{h}}}_{\mathbf{p}}} \right)} \right],$$

$${\mathbf{W}}_{{{\mathbf{v}},{\mathbf{c}}}} = {\text{diag}}\left[ {\frac{1}{2}\left( {1 - {{\mathbf{h}}}_{\mathbf{c}}} \right)^{2} \left( {\frac{{\hat{\sigma }_{{e_{a} }}^{2} }}{{\hat{\sigma }_{{e_{s} }}^{2} }}} \right)^{2} } \right],$$

i.e. the reciprocals of the predicted residual variance from the previous iteration and, $$\sigma_{{\varepsilon_{p} }}^{2} ,$$$$\sigma_{{\varepsilon_{c} }}^{2} ,$$$$\sigma_{{\varepsilon_{{v_{p} }} }}^{2}$$ and $$\sigma_{{\varepsilon_{{v_{c} }} }}^{2}$$ are the scaling variances, which are expected to be equal to 1, since $${\mathbf{W}}_{{\mathbf{p}}}$$, $${\mathbf{W}}_{{\mathbf{c}}}$$, $${\mathbf{W}}_{{{\mathbf{v}},{\mathbf{p}}}}$$ and $${\mathbf{W}}_{{{\mathbf{v}},{\mathbf{c}}}}$$ already contained the reciprocals of the predicted residual variances per observation [18]. The adjustment in $${\mathbf{W}}_{{\mathbf{c}}}$$ for crossbreds was to account for the fact that only the $$V_{\text{E}}$$ part of the residual variance was heterogeneous; the adjustment in $${\mathbf{W}}_{{{\mathbf{v}},{\mathbf{c}}}}$$ was based on the derivation in Mulder et al. [18]. The vectors $${\mathbf{h}}_{\mathbf{p}}$$ and $${\mathbf{h}}_{\mathbf{c}}$$ contained the leverage for each purebred and crossbred observation. The model for purebreds was equivalent to Felleki et al. [17]; the model for crossbreds was equivalent to Mulder et al. [18], without the slope of the reaction norm. The method required a number of ASReml runs to update the response variables $${\mathbf{y}}_{{{\mathbf{v}}_{{\mathbf{p}}} }}$$ and $${\mathbf{y}}_{{{\mathbf{v}}_{{\mathbf{c}}} }}$$ and the matrices $${\mathbf{W}}_{{\mathbf{p}}}$$, $${\mathbf{W}}_{{\mathbf{c}}}$$, $${\mathbf{W}}_{{{\mathbf{v}},{\mathbf{p}}}}$$ and $${\mathbf{W}}_{{{\mathbf{v}},{\mathbf{c}}}}$$. The initial values of residual variance for DHGLM analyses were taken from the model assuming homogeneous residual variance.

The algorithm for the iterations was as follows [17, 18]:

1. Run linear mixed model for ${\mathbf{y}}_{{\mathbf{p}}}$ and ${\mathbf{y}}_{{\mathbf{c}}}$ with homogeneous residual variance.
2. Calculate $${\mathbf{y}}_{{{\mathbf{v}}_{{\mathbf{p}}} }}$$, $${\mathbf{y}}_{{{\mathbf{v}}_{{\mathbf{c}}} }}$$, $${\mathbf{W}}_{{\mathbf{p}}}$$, $${\mathbf{W}}_{{\mathbf{c}}}$$, $${\mathbf{W}}_{{{\mathbf{v}},{\mathbf{p}}}}$$ and $${\mathbf{W}}_{{{\mathbf{v}},{\mathbf{c}}}} ,$$
  where $${\mathbf{W}}_{{\mathbf{p}}} = {\text{diag}}\left( {\frac{1}{{\sigma_{{e_{p} }}^{2} }}} \right)$$ and $${\mathbf{W}}_{{\mathbf{c}}} = {\text{diag}}\left( {\frac{1}{{\sigma_{{e_{c} }}^{2} }}} \right),$$
  and where $\sigma_{{e_{p} }}^{2}$ and $\sigma_{{e_{c} }}^{2}$ are the residual variances in the first iteration. Note that there was an error in Mulder et al. [18] where the residual variance was used in ${\mathbf{W}}$ instead of the reciprocal of the residual variance.
3. Run a four-variate linear mixed model on ${\mathbf{y}}_{{\mathbf{p}}}$, ${\mathbf{y}}_{{\mathbf{c}}}$, ${\mathbf{y}}_{{{\mathbf{v}}_{{\mathbf{p}}} }}$ and ${\mathbf{y}}_{{{\mathbf{v}}_{{\mathbf{c}}} }}$.
4. Update $${\mathbf{y}}_{{{\mathbf{v}}_{{\mathbf{p}}} }}$$, $${\mathbf{y}}_{{{\mathbf{v}}_{{\mathbf{c}}} }}$$, $${\mathbf{W}}_{{\mathbf{p}}}$$, $${\mathbf{W}}_{{\mathbf{c}}}$$, $${\mathbf{W}}_{{{\mathbf{v}},{\mathbf{p}}}}$$ and $${\mathbf{W}}_{{{\mathbf{v}},{\mathbf{c}}}}$$.
5. Iterate steps 3 and 4 until convergence.

The algorithm was run for 100 iterations and parameters showed small changes. The sum of the relative squared differences in estimated values of all variance components between the current and the previous iteration was between 3 × 10^−3^ and 1 × 10^−2^ for the iterations 51 to 100. In addition, individual parameters showed only minor changes (<5 %). Therefore, we considered that the algorithm converged after 100 iterations.

### Estimating genetic correlations between periods

In Eq. 1, a repeatability model was used assuming that eggshell color was genetically the same trait across the whole laying period. Eggs of purebred laying hens were measured during four laying periods and eggs of crossbred laying hens were measured during three laying periods (see Section “Data”). Therefore, bivariate analyses were done to estimate variance components for these different periods and to estimate genetic correlations between periods. We used the final weights and response variables ${\mathbf{y}}_{{{\mathbf{v}}_{{\mathbf{p}}} }}$, ${\mathbf{y}}_{{{\mathbf{v}}_{{\mathbf{c}}} }}$, ${\mathbf{W}}_{{\mathbf{p}}}$, ${\mathbf{W}}_{{\mathbf{c}}}$, ${\mathbf{W}}_{{{\mathbf{v}},{\mathbf{p}}}}$ and ${\mathbf{W}}_{{{\mathbf{v}},{\mathbf{c}}}}$ from Eq. 1 and used Eq. 1 on subsets of data corresponding to the periods mentioned. The model included the same fixed and random effects as Eq. 1. Note that for the bivariate analyses that involved only laying periods for purebreds, the cage effect was replaced by a permanent environmental effect for the second period and for the bivariate analyses that involved only laying periods for crossbreds, the permanent environmental effect was replaced by a cage effect for the first period. Unfortunately, the analyses between different laying periods of purebred and crossbred laying hens and among laying periods in crossbred laying hens did not converge or had very large standard errors. Therefore, only genetic correlations between different laying periods in purebred laying hens are presented in the “Results” section ‘Genetic correlations between different laying periods’.

### Effect of different definitions of environmental variance

As described earlier, the definition of $V_{\text{E}}$ differed between purebreds and crossbreds. In purebreds, $V_{\text{E}}$ was the within-individual variance of eggshell color, because repeated observations per hen were available. In crossbreds, $V_{\text{E}}$ contained both within-individual variance and between-hen variance. This difference in definition may affect the size of the genetic variance in $V_{\text{E}}$ and the genetic correlation between $V_{\text{E}}$ in purebreds and crossbreds. To investigate the effect of this difference in the definition of $V_{\text{E}}$, we performed 20 replicates using purebred data for which half of the daughters of each sire was randomly assigned to individual cages and the other half to multiple-hen cages that contained four hens to mimic the situation of the purebred and crossbred laying hens. We used the model in Eq. 1, except that the fixed effects were only hatch week and laying date. The main parameters were the genetic variances in $V_{\text{E}}$ in ‘individual cages’ and ‘multiple-hen cages’, and the genetic correlation between $V_{\text{E}}$ in ‘multiple-hen cages’ and $V_{\text{E}}$ in ‘individual cages’. From these analyses and the estimated genetic correlation between $V_{\text{E}}$ in purebred and crossbred laying hens, we back-calculated the genetic correlation between $V_{\text{E}}$ in purebred and crossbred laying hens when the definition of $V_{\text{E}}$ would have been the same, i.e. the within-individual variance (see “Appendix” section). This calculation provided insight into the extent to which the estimated genetic correlation between $V_{\text{E}}$ in purebred and crossbred laying hens was due to a difference in definition of $V_{\text{E}}$.

### Calculation of genetic parameters

In order to compare our results with data in the literature, we calculated two additional genetic parameters based on the estimated variance components, i.e. the heritability of $V_{\text{E}}$ at the individual record level ($h_{v}^{2}$) and the genetic coefficient of variation for $V_{\text{E}}$ ($GCV_{Ve}$) [4]. The $h_{v}^{2}$ can be used to calculate the accuracy of selection and $GCV_{Ve}$ indicates how much $V_{\text{E}}$ can be changed by selection [20]. The $h_{v}^{2}$ is defined as the regression of the breeding value for $V_{\text{E}}$ on the squared phenotypic deviation as an analogy of the normal heritability using an additive model for $V_{\text{E}}$ [20]. The calculation was done following [21, 22], for details see the Appendix in [21]. The $GCV_{Ve}$ was calculated as:

2 $$GCV_{Ve} = \sigma_{{a_{v} }}$$

Standard errors of $h_{v}^{2}$ and $GCV_{Ve}$ were calculated using Taylor series approximations. Derivations are shown in the “Appendix”. Fortran code is provided in Additional file 1.

## Results

### Summary of the phenotypic data

**Fig. 1:**
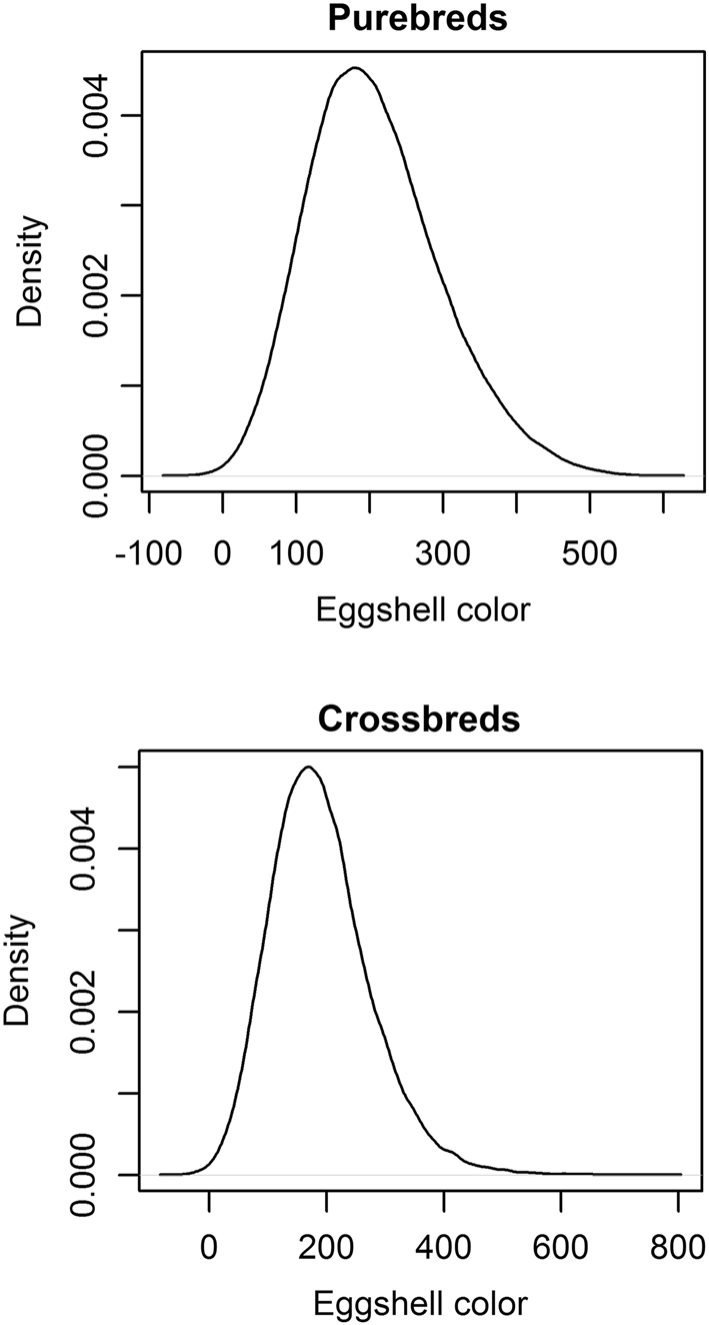
Distributions of eggshell color in purebred and crossbred laying hens

Eggshell color was approximately normally distributed with small skewness and kurtosis (Table 1; Fig. 1). The deviation from normality was slightly greater for crossbred laying hens. Means and standard deviations were similar for eggs of purebred and crossbred laying hens.

### Genetic variation in eggshell color and its environmental variance

The variance components for eggshell color itself are in Table 2 and for $V_{\text{E}}$ in Table 3. Heritabilities of 0.32 and 0.39 were found for eggshell color of purebreds and crossbreds, respectively. For purebreds, permanent environmental effects explained a large proportion of the phenotypic variance even after subtracting three quarters of the genetic variance (16.7 %), i.e. the additive genetic variance due to dam and Mendelian sampling. For crossbreds, cage explained a relatively small proportion of the variance (5.2 %). For $V_{\text{E}}$, genetic coefficients of variation ranged from 0.26 to 0.28, which indicates that $V_{\text{E}}$ could be changed by 26 to 28 % when changing $V_{\text{E}}$ with one genetic standard deviation. Heritabilities for $V_{\text{E}}$ ($h_{v}^{2}$) were equal to 0.01. Standard errors on estimated variance components and derived parameters were small. These estimated genetic variances in $V_{\text{E}}$ of eggshell color in purebred and crossbred laying hens indicate that there are opportunities for genetic improvement of uniformity.

**Table 2:** Variance components for eggshell color in purebred and crossbred laying hens

Variance component	Purebred	Crossbred
Sire	550.0 (51.5)	602.2 (38.2)
Permanent environment	2803.0 (34.0)	
Cage		339.0 (15.9)
Residual^a^	3547.0 (13.0)	5583.0 (28.9)
Genetic	2200.0 (206.0)	2408.8 (152.7)
*h* ^2b^	0.32 (0.028)	0.37 (0.022)

Standard errors are listed between brackets

^a^Residual variance was taken from the model with homogeneous residual variance

^b^The standard error of the *h* ^2^ was based on the model with homogeneous residual variance

**Table 3:** Variance components for the environmental variance (exponential model) of eggshell color in purebred and crossbred laying hens

Variance component	Purebred	Crossbred
Sire	0.019 (0.003)	0.017 (0.005)
Permanent environment	0.32 (0.006)	
Cage		0.098 (0.010)
Residual^a^	1.847 (0.007)	5.786 (0.030)
Genetic	0.077 (0.011)	0.067 (0.020)
$h_{v}^{2}$ ^b^	0.010 (0.001)	0.011 (0.003)
*GCV*_*Ve*_^b^	0.277 (0.019)	0.259 (0.039)

Standard errors are provided between brackets

^a^The residual variance for purebreds is lower than in crossbreds due to sire model adjustment in crossbreds

^b^Approximate standard errors were calculated according to formulae in the “Appendix”

### Genetic correlations between purebreds and crossbreds

The genetic correlation between purebred and crossbred eggshell color was equal to 0.86 (Table 4), which indicates that eggshell color is genetically very similar in purebreds and crossbreds. For $V_{\text{E}}$, the genetic correlation was equal to 0.70 and indicated that $V_{\text{E}}$ in purebreds and crossbreds is genetically similar but more different than eggshell color itself. Genetic correlations between eggshell color and $V_{\text{E}}$ were about zero in purebreds and positive in crossbreds. Covariances between eggshell color and $V_{\text{E}}$ were significantly different between purebreds and crossbreds (p < 0.001; two-sided *t* test, approximate test assuming normality of the test statistic [23]). In purebreds, selection for a lower eggshell color score (darker brown eggs) does not change $V_{\text{E}}$, while in crossbreds, selection for a lower eggshell color (darker brown eggs) results in a lower $V_{\text{E}}$, i.e. higher uniformity.

**Table 4:** Genetic correlations between eggshell color and its environmental variance in purebred and crossbred laying hens

Trait	Trait		
	Eggshell color	Environmental variance	
	Crossbred	Purebred	Crossbred
Eggshell color			
Purebred	0.86 (0.047)	−0.057 (0.084)	0.19 (0.15)
Crossbred		−0.013 (0.11)	0.43 (0.10)
Environmental variance			
Purebred			0.70 (0.19)

Standard errors are provided between brackets

### Genetic correlations between different laying periods

In purebreds, we investigated the genetic correlations between different laying periods in purebred laying hens (Table 5). The genetic variance for $V_{\text{E}}$ was smallest in the early laying period, whereas it was approximately constant in mid and late laying periods. Genetic correlations between periods were higher than 0.86, except between periods 1 (25 to 35 weeks of age) and 3 (56 to 75 weeks of age) and between periods 1 (25 to 35 weeks of age) and 4 (76 to 95 weeks of age). This indicates that $V_{\text{E}}$ is approximately the same trait across laying periods, except for the early laying period.

**Table 5:** Genetic parameters for environmental variance of eggshell color in different periods in purebred laying hens

Period	Period			
	1	2	3	4
1	0.067 (0.014)	0.92 (0.062)	0.68 (0.095)	0.64 (0.139)
2		0.092 (0.015)	0.89 (0.051)	0.86 (0.074)
3			0.088 (0.015)	0.94 (0.059)
4				0.084 (0.019)

There were 33,395, 50,919, 52,682 and 30,655 records in periods 1, 2, 3 and 4, respectively

Periods 1 = early, 2 = mid 1, 3 = mid 2 and 4 = late laying period; genetic variances on diagonal, upper off-diagonals are genetic correlations; standard errors are listed between brackets

### Effect of different definitions of environmental variance

The results of simulations to test the effect of different definitions of $V_{\text{E}}$ are in Table 6. The genetic correlation between individual cages and multiple-hen cages for $V_{\text{E}}$ was equal to 0.73, i.e. slightly higher than the genetic correlation between purebreds and crossbreds for $V_{\text{E}}$. If the definition of $V_{\text{E}}$ for purebreds and crossbreds had been identical, i.e. the within-individual variance based on individual cages, then the genetic correlation between $V_{\text{E}}$ in purebreds and crossbreds would have been equal to 0.95 using Eq. 17 of the “Appendix”. Furthermore, we found that the genetic variance in $V_{\text{E}}$ (0.14) almost doubled for multiple-hen cages compared to individual cages (0.077) and in crossbreds (0.067). This seems to indicate that the between-individual component of $V_{\text{E}}$ may have a genetic component. The genetic variance of the between-individual component of $V_{\text{E}}$ was equal to 0.064, using Eq. 16 of the “Appendix”, which was almost as large as the genetic variance in $V_{\text{E}}$ for the within-individual component of $V_{\text{E}}$, e.g. in purebreds that were in individual cages. Furthermore, using Eq. 15 (“Appendix”), the genetic correlation between within-individual and between-individual components of $V_{\text{E}}$ was equal to −0.01, which indicates that these two parts of $V_{\text{E}}$ were genetically different traits. These simulations show that the deviation from 1 of the correlation between purebreds and crossbreds was mainly caused by the difference in definition of $V_{\text{E}}$ for individually-housed purebred hens and crossbred hens housed in multiple-hen cages. The correlation between purebreds and crossbreds is proportional to the square root of the ratio of the within-individual component of $V_{\text{E}}$ and the sum of within-individual and between-individual components of $V_{\text{E}}$ assuming that the genetic correlation between both components is zero (“Appendix”).

**Table 6:** Genetic variance in environmental variance in multiple-hen cages and the genetic correlations with individual cages for eggshell color

Parameter	Crossbred	Purebred simulations	
		Mean	SD
Genetic variance	0.067	0.14	0.015
Genetic correlation for eggshell color	0.86	0.93	0.018
Genetic correlation for environmental variance different definitions^a^	0.70	0.73	0.10
Genetic correlation for environmental variance equal definition^b^	0.95	1.00	–

Comparison of 20 replicates (mean and standard deviation) between purebreds and crossbreds

^a^For purebreds, in individual cages, the environmental variance contains only within-individual variance, whereas the environmental variance in multiple-hen cages (also crossbreds) contains within-individual variance and between-individual variance, i.e. different definitions of environmental variance

^b^Here the interest lies in the genetic correlation between individual variance in purebred and crossbred laying hens, i.e. equal definition of environmental variance. Therefore, the expected genetic correlation in the purebred simulations is 1.00. Based on this assumption, Eq. 17 can be used to calculate the estimated genetic correlation between within-individual variance in purebred and crossbred laying hens

## Discussion

### Genetic variance in uniformity

In this study, we estimated the genetic variance in $V_{\text{E}}$ of eggshell color in purebred and crossbred laying hens as well as the genetic correlations between $V_{\text{E}}$ in purebred and crossbred laying hens and between $V_{\text{E}}$ in different laying periods. The DHGLM methodology was extended to a bivariate version to analyze eggshell color and its $V_{\text{E}}$ as separate traits in purebred and crossbred laying hens.

To the best of our knowledge, this paper reports the first estimates of genetic variance for $V_{\text{E}}$ of eggshell color in purebred and crossbred laying hens. Estimates in purebreds and crossbreds were similar and slightly higher in purebreds than in crossbreds. The genetic coefficient of variation ($GCV_{Ve}$) was close to the median value found for other traits in other species [4]. The heritability of $V_{\text{E}}$ was low, but comparable to those reported in other recent studies [5, 18, 24]. The low heritability indicates that large volumes of data are needed to obtain accurate breeding values for $V_{\text{E}}$. It should be noted that the heritability is at the individual record level and therefore estimating a breeding value for $V_{\text{E}}$ based on a single observation is not accurate. For instance, according to Tukey’s rule, estimating variances with the same accuracies as for the means requires five times more observations [25]. With repeated observations, alternatively one can analyze the log variance or the standard deviation of egg color, similar to Wolc et al. [7]. When performing a genetic analysis using the log variance in purebreds, a genetic variance of 0.097 and a heritability of 0.15 were found. Due to the use of the log variance, the estimate of the genetic variance can be compared to the estimate from DHGLM, because both assume an exponential model for $V_{\text{E}}$ [5]. The heritability estimate of 0.15 is low to moderate and comparable to the heritability of number of eggs produced during a 2-week period in the first month of egg production [26]. This simple analysis shows good prospects for the estimation of EBV for $V_{\text{E}}$. The difference in heritabilities between the DHGLM and the simple analysis is due to the difference in trait definition: the trait definition used in the DHGLM is based on the individual record level, whereas that in the simple analysis is based on the log-variance of about 10 repeated observations. Both analyses gave similar estimates of genetic variance, but a very different view on the heritability. Note that the DHGLM is better capable of adjusting for systematic environmental effects such as the day of egg laying than the simple method and will yield similar accuracies of EBV [5]. Thus, we advocate that the heritability on the individual record level should be used only to calculate the accuracy of selection, otherwise it may give a misleading judgment on the size of the genetic variance. From evolutionary genetics, we know that the heritability is a poor predictor for response to selection, because it does not directly indicate how much the trait mean can be changed by selection [27, 28]. Therefore, one needs to know how large the genetic variation is relative to the trait mean, i.e. the genetic coefficient of variation (GCV) ($\sigma_{A} /\mu$) [28]. To interpret the size of the genetic variance in $V_{\text{E}}$, we recommend the use of $GCV_{Ve}$, because it gives an indication of the potential response to selection in $V_{\text{E}}$. For instance, if the response to selection is one genetic standard deviation downward (e.g. selection intensity is 2.0 and accuracy is 0.5), than $V_{\text{E}}$ is reduced by 26 to 28 % if $GCV_{Ve}$ is equal to 26 to 28 %.

### The DHGLM model

For crossbreds, we used the sire model adjustment [18] to account for the fact that the residual variance contains three-quarters of the genetic variance of eggshell color itself. Simulations showed that standard DHGLM would underestimate the genetic variance in $V_{\text{E}}$ and the proposed adjustment resulted in unbiased estimates of genetic variance [18]. In this study, when we used the standard DHGLM, the genetic variance in $V_{\text{E}}$ was indeed less than with the adjusted DHGLM, but the difference in estimates was smaller than theoretically expected. This may indicate that the Mendelian sampling variance is heterogeneous between sires. Disentangling Mendelian sampling variance and $V_{\text{E}}$ is, however, impossible for the crossbred data in this dataset. Although the genetic variance changed when using either standard DGHLM or adjusted DHGLM, the estimated genetic correlation between $V_{\text{E}}$ in purebred and crossbred layer hens was the same.

The genetic analysis that considered the different laying periods as separate traits revealed that except for the early laying period, eggshell color and its $V_{\text{E}}$ are genetically very similar traits across the whole egg laying period. Thus, except for the early laying period, a repeatability model seems justified for the other later laying periods. Random regression models such as test-day models [29, 30] could be used to model with greater flexibility the genetic variance–covariance structure along the laying period. It should be noted that such models are much more demanding and the increase in accuracy is probably limited.

### The definition of environmental variance

Based on the simulations in purebreds, we concluded that the genetic correlation between $V_{\text{E}}$ in purebred and crossbred laying hens ($r_{pc}$) deviated from 1 mainly because of a difference in definition of $V_{\text{E}}$. Surprisingly, the genetic variance in $V_{\text{E}}$ was almost doubled when analyzing the purebred data as if they were in multiple-hen cages. This indicated that some genetic variance in the between-individual variance contributed to $V_{\text{E}}$. Because in our simulations, we used records on purebred laying hens that were individually housed, the between-individual variance was due to differences in permanent environmental effects and the non-explained additive and non-additive genetic differences between individuals. In [4], a genetic model for both genetic differences in $V_{\text{E}}$ and the permanent environmental variance was postulated, although no scientific evidence was available at that time. To our knowledge, these results suggest, for the first time, the existence of a genetic component in the between-individual variance of $V_{\text{E}}$. Although, we observed an increase in genetic variance in $V_{\text{E}}$ when assuming that the purebreds were in multiple-hen cages, we did not observe such an increase in genetic variance in $V_{\text{E}}$ between crossbreds and purebreds. This may suggest that the between-individual component of $V_{\text{E}}$ in crossbreds is different from that in purebreds, e.g. that it is more related to interactions between hens rather than differences in permanent environmental variance. For instance, within-individual and between-individual components of $V_{\text{E}}$ may be negatively correlated and thus there would be no increase in genetic variance in $V_{\text{E}}$ (see “Appendix” for the genetic model). From a scientific point of view, it is interesting to disentangle the genetic correlation between purebreds and crossbreds that is partly due to a difference in definition of $V_{\text{E}}$ and partly due to the genetic correlation between within-individual variance in purebreds and crossbreds. These simulations in purebreds not only show the need for a proper definition of $V_{\text{E}}$, but also that it might be interesting to study the genetics of the between-individual component of $V_{\text{E}}$. Furthermore, from a breeding goal point of view, increasing uniformity of eggs between hens is as important as improving uniformity within hens. However, no statistical methodology is available to estimate genetic variance for the between-hen (effectively the permanent environment effect) and the within-hen component of $V_{\text{E}}$ and therefore the back-calculation method as described in the last section of the “Appendix” was used to provide insight into the contributions of both components.

### Estimation of genetic correlations between purebreds and crossbreds for uniformity

To the best of our knowledge, this is the first time that genetic correlations between purebred and crossbred laying hens for $V_{\text{E}}$ and genetic correlations between $V_{\text{E}}$ for different laying periods are reported. The genetic correlation between purebred and crossbred performance ($r_{pc}$) is the key parameter that determines the need for crossbred information in purebred selection when crossbred performance is the breeding goal [14]. In our study, we found an $r_{pc}$ of 0.86 for eggshell color and 0.70 for $V_{\text{E}}$. One might expect $r_{pc}$ to be very similar for eggshell color and $V_{\text{E}}$. In addition to the difference in definition of $V_{\text{E}}$, the lower $r_{pc}$ for $V_{\text{E}}$ might be due to $V_{\text{E}}$ being more sensitive to genotype-by-environment interaction than eggshell color itself. Purebreds are housed in a highly hygienic nucleus environment, whereas crossbreds are kept in a production environment. Therefore, crossbreds are likely to be more challenged by environmental disturbances such as diseases. These differences in environment may contribute to a genotype-by-environment interaction component in the estimate of $r_{pc}$ and may affect $V_{\text{E}}$ more than eggshell color itself.

Designs to estimate $r_{pc}$ for $V_{\text{E}}$ require large amounts of data due to the low heritability of $V_{\text{E}}$. The equation to approximate the standard error for $r_{pc}$ presented by Bijma and Bastiaansen [14] was used to search for designs that result in a standard error as low as 0.1 when $r_{pc}$ = 0.7 and $h_{v}^{2}$ = 0.01, ignoring cage or permanent environmental effects. With 500 sire families, approximately 270 purebred and crossbred offspring per family are required for traits that are measured only once, whereas with 200 sire families, about 500 purebred and crossbred offspring per family are required. Thus large datasets with more than 200,000 records would be needed. Therefore, for traits that are measured only once per animal, such as growth rate in pigs, it might be challenging to obtain such large data sets. Fortunately, for such traits $h_{v}^{2}$ seems larger [4, 31]. When the $h_{v}^{2}$ is equal to 0.03 instead of 0.01, about 170 purebred and crossbred offspring from 200 sire families are required alleviating the requirements on the size and structure of the dataset. With repeated observations such as eggshell color, fewer offspring per family are required. With 10 repeated observations, approximately 60 purebred and 60 crossbred offspring per sire are required with 200 sire families. It can be concluded that for estimating $r_{pc}$ for $V_{\text{E}}$, very large datasets are needed.

In this study, the DHGLM methodology was used to estimate the genetic correlation between $V_{\text{E}}$ in purebreds and crossbreds, but the same methodology can be used to estimate the genetic correlation between $V_{\text{E}}$ in different environments to investigate genotype-by-environment interactions. In a previous study [21], we investigated $V_{\text{E}}$ for fish raised in fresh and seawater and found genotype-by-environment interactions for $V_{\text{E}}$, especially after log-transforming the data. Due to different micro-environmental factors in these environments, genotype-by-environment interactions for $V_{\text{E}}$ may arise. The method of Bijma and Bastiaansen [14] can be used to design experiments or to evaluate how datasets should be created to estimate genotype-by-environment interactions for $V_{\text{E}}$.

### Implications for breeding

The estimates of genetic variance for $V_{\text{E}}$ found in this study are encouraging for the genetic improvement of uniformity of eggshell color. From a trait point of view, there is probably more interest in improving uniformity than in changing eggshell color itself. The breeding goal is to have dark brown eggs with high uniformity. This means that the eggshell color index should have low values and little variation. Furthermore, eggshell color should not change too much during the whole laying period. Recurrent testing is common practice in laying hens and crossbred information will increase the accuracy of selection, especially for males. Although estimates of $r_{pc}$ are high, combined crossbred and purebred selection is expected to result in a higher response to selection than purebred selection [32], but also to increased costs of recording. When using standard selection index equations to predict the accuracy of EBV with a single source of information, the accuracy of purebred females based on 10 own repeated observations would be equal to 0.27. For sires, an accuracy of about 0.7 would be found when measuring about 500 eggs of half-sib offspring and about 0.8 when measuring 1000 eggs. If the best 15 % of the sires are selected with an accuracy of 0.7 and the best 20 % of the hens with an accuracy of 0.27 and $GCV_{Ve}$ = 0.28, the selection response would lead to a reduction of 19 % in $V_{\text{E}}$ and 10 % in $V_{\text{P}}$ (Table 2) after one generation of selection, which opens up good prospects for selection on uniformity in agreement with earlier studies [20, 33]. Such selection would increase the uniformity of eggs; in other words, the frequency of extremely dark brown eggs or white eggs would be lower. Because of the positive genetic correlation between eggshell color and its $V_{\text{E}}$ in crossbred laying hens, selection on uniformity would yield darker brown eggs because the eggshell color value would decrease as a correlated response.

In addition to selection on uniformity in the pure lines, uniformity at the producer level could be achieved by selecting sires and dams as parents for the crossbreds on their EBV for $V_{\text{E}}$. Furthermore, one could select sires and dams with minimal genetic differences in eggshell color, i.e. similar EBV for eggshell color itself. It should be noted, however, that offspring still show genetic variation in eggshell color due to prediction error variance of EBV and Mendelian sampling. However, selection on lower $V_{\text{E}}$ in pure lines is favored, because it would result in a permanent increase in uniformity of eggshell color in purebreds and crossbreds.

## Conclusions

 The genetic coefficients of variation for $V_{\text{E}}$ of eggshell color in purebred and crossbred laying hens ranged from 26 to 28 %. The genetic correlation between purebred and crossbred $V_{\text{E}}$ of eggshell color was 0.70. The deviation from 1 of this genetic correlation is mainly due to a difference in the definition of $V_{\text{E}}$ between purebred and crossbred hens. This indicates that there is some reranking of sires for $V_{\text{E}}$ of eggshell color in purebred and crossbred laying hens. Genetic correlations between $V_{\text{E}}$ of eggshell color in different laying periods were generally higher than 0.85, except between early laying and mid or late laying periods. The results indicate that there are good opportunities to improve uniformity of eggshell color in purebreds and crossbreds by genetic selection, ideally with combined crossbred and purebred selection. The methodology that we developed here can be used to estimate genetic correlations between purebreds and crossbreds for uniformity of other traits or species such as pigs.

## Appendix: Approximate standard errors for derived genetic parameters $\varvec{h}_{\varvec{v}}^{2}$ and $\varvec{GCV}_{{\varvec{Ve}}}$

Approximate standard errors for $h_{v}^{2}$ and $GCV_{Ve}$ were derived using Taylor series approximations as shown in Lynch and Walsh [23]. Because $h_{v}^{2}$ is a ratio [20, 21], we derive the sampling variance of the nominator and the denominator and subsequently the sampling variance of the ratio of the nominator and denominator. The nominator of $h_{v}^{2}$ is the additive genetic variance for $V_{\text{E}}$ on the additive scale $\sigma_{{a_{v,add} }}^{2}$. We ignored the sampling variance on $\sigma_{{E_{exp} }}^{2}$, because its relative standard error is small compared to the relative standard error of $\sigma_{{a_{v} }}^{2}$ and therefore the contribution to the sampling variance of $\sigma_{{a_{v,add} }}^{2}$ is negligible. Therefore, considering $\sigma_{{E_{exp} }}^{2}$ as a constant, the sampling variance of $\sigma_{{a_{v,add} }}^{2} + \sigma_{{c_{v,add} }}^{2}$ can be approximated using equation A1.7c in Lynch and Walsh [23], where $\sigma_{{c_{v} }}^{2}$ is $\sigma_{{pe_{{v_{p} }} }}^{2}$ for purebreds and $\sigma_{{cg_{{v_{c} }} }}^{2}$ for crossbreds. Assuming no sampling covariance between $\sigma_{{a_{v,add} }}^{2}$ and $\sigma_{{c_{v,add} }}^{2}$, the sampling variance of $\sigma_{{a_{v,add} }}^{2} + \sigma_{{c_{v,add} }}^{2}$ can be split up into a part due to $\sigma_{{a_{v,add} }}^{2}$ and a part due to $\sigma_{{c_{v,add} }}^{2}$. Equation 3 shows the sampling variance for $\sigma_{{a_{v,add} }}^{2}$ ($var\sigma_{{a_{v} }}^{2}$):

3 $$\begin{aligned} var\sigma_{{a_{v,add} }}^{2} & = {\text{var}}\left(\sigma_{{E_{exp} }}^{4} \exp \left( {2\sigma_{{a_{v} }}^{2} } \right)\right) + {\text{var}}\left( {\sigma_{{E_{exp} }}^{4} \exp \left( {\sigma_{{a_{v} }}^{2} } \right)} \right) \\ & \quad - 2{\text{cov}}\left(\sigma_{{E_{exp} }}^{4} \exp \left( {2\sigma_{{a_{v} }}^{2} } \right),\sigma_{{E_{exp} }}^{4} \exp \left( {\sigma_{{a_{v} }}^{2} } \right)\right) \\ var\sigma_{{a_{v,add} }}^{2} & \cong \sigma_{{E_{exp} }}^{8} { \exp }\left( {2\sigma_{{c_{v} }}^{2} } \right)var\sigma_{{a_{v} }}^{2} \left( {4\exp \left( {4\sigma_{{a_{v} }}^{2} } \right) - 4\exp \left( {3\sigma_{{a_{v} }}^{2} } \right) + { \exp }\left( {2\sigma_{{a_{v} }}^{2} } \right)} \right). \\ \end{aligned}$$

The denominator of $h_{v}^{2}$ is $2\sigma_{P}^{4} + 3\left( {\sigma_{{a_{v,add} }}^{2} + \sigma_{{c_{v,add} }}^{2} } \right)$. When ignoring sampling covariances, the sampling variance of the denominator is:

4 $$\begin{aligned}& {\text{var}}\left(2\sigma_{P}^{4} + 3\left( {\sigma_{{a_{v,add} }}^{2} + \sigma_{{c_{v,add} }}^{2} } \right)\right) \hfill \\ &\quad = {\text{var}}(2\sigma_{P}^{4} ) + 9\left( {var\sigma_{{a_{v,add} }}^{2} + var\sigma_{{c_{v,add} }}^{2} } \right). \hfill \\ \end{aligned}$$

When using the variance of a product in equation A1.18b in Lynch and Walsh [23]:

5 $${\text{var}}(2\sigma_{P}^{4} ) = 8*\left( {\left( {2*\sigma_{P}^{4} *var\sigma_{P}^{2} } \right) + \left( {var\sigma_{P}^{2} } \right)^{2} } \right)$$

Similar to Eq. 3:

6 $$var\sigma_{{c_{v,add} }}^{2} \cong \sigma_{{E_{exp} }}^{8} { \exp }\left( {2\sigma_{{a_{v} }}^{2} } \right)var\sigma_{{c_{v} }}^{2} \left( {4\exp \left( {4\sigma_{{c_{v} }}^{2} } \right) - 4\exp \left( {3\sigma_{{c_{v} }}^{2} } \right) + { \exp }\left( {2\sigma_{{c_{v} }}^{2} } \right)} \right)$$

Combining Eqs. 4, 5 and 6 gives:

7 $$\begin{aligned} var_{denom} & = {\text{var}}(2\sigma_{P}^{4} + 3\left( {\sigma_{{a_{v,add} }}^{2} + \sigma_{{c_{v,add} }}^{2} } \right)) = 8*\left( {\left( {2*\sigma_{P}^{4} *var\sigma_{P}^{2} } \right) + \left( {var\sigma_{P}^{2} } \right)^{2} } \right) \\ & \quad + 9(\sigma_{{E_{exp} }}^{8} var\sigma_{{a_{v} }}^{2} \left( {4\exp \left( {4\sigma_{{a_{v} }}^{2} } \right) - 4\exp \left( {3\sigma_{{a_{v} }}^{2} } \right) + \exp \left( {2\sigma_{{a_{v} }}^{2} } \right)} \right) \\ & \quad + \sigma_{{E_{exp} }}^{8} var\sigma_{{c_{v} }}^{2} \left( {4\exp \left( {4\sigma_{{c_{v} }}^{2} } \right) - 4\exp \left( {3\sigma_{{c_{v} }}^{2} } \right) + { \exp }\left( {2\sigma_{{c_{v} }}^{2} } \right)} \right) \\ \end{aligned}$$

Subsequently, the sampling variance of $h_{v}^{2}$ is approximated with equation A1.19b in Lynch and Walsh [23], assuming that:

8 $${\text{cov}}\left( {\sigma_{{a_{v,add} }}^{2} ,2\sigma_{P}^{4} + 3\left( {\sigma_{{a_{v,add} }}^{2} + \sigma_{{c_{v,add} }}^{2} } \right)} \right) = 3var\sigma_{{a_{v,add} }}^{2} \hfill$$

9 $$ varh_{v}^{2} \cong h_{v}^{4} \left[ {\frac{{var\sigma_{{a_{v,add} }}^{2} }}{{\sigma_{{a_{v,add} }}^{4} }} - \frac{{6var\sigma_{{a_{v,add} }}^{2} }}{{\sigma_{{a_{v,add} }}^{2} *\left[ {2\sigma_{P}^{4} + 3\left( {\sigma_{{a_{v,add} }}^{2} + \sigma_{{c_{v,add} }}^{2} } \right)} \right]}} + \frac{var\_denom}{{\left[ {2\sigma_{P}^{4} + 3\left( {\sigma_{{a_{v,add} }}^{2} + \sigma_{{c_{v,add} }}^{2} } \right)} \right]^{2} }}} \right] \hfill$$

The standard error of $h_{v}^{2}$ is then:

10 $$seh_{v}^{2} = \sqrt {varh_{v}^{2} }$$

Numerical analysis showed that $varh_{v}^{2} \cong h_{v}^{4} \frac{{var\sigma_{{a_{v,add} }}^{2} }}{{\sigma_{{a_{v,add} }}^{4} }}$, which indicates that the last two terms in Eq. 8 are mostly cancelling out each other.

The standard error of $GCV_{Ve}$ was approximated using equation A1.7c in Lynch and Walsh [23]:

11 $$varGCV_{Ve} \cong var\left( {\sqrt {\sigma_{{a_{v} }}^{2} } } \right) = var\sigma_{{a_{v} }}^{2} /4\sigma_{{a_{v} }}^{2}$$

12 $$seGCV_{Ve} \cong se\sigma_{{a_{v} }}^{2} /2\sigma_{{a_{v} }}^{ }$$

## Contribution of the difference in definition of $\varvec{V}_{{\mathbf{E}}}$ to the genetic correlation between purebred and crossbred $\varvec{V}_{{\mathbf{E}}}$

Purebred hens were in individual hen cages and crossbred hens were in multiple-hen cages. This difference in housing led to a difference in the definition of $V_{\text{E}}$. The aim here was to investigate the contribution of the difference in definition of $V_{\text{E}}$ to the genetic correlation between purebred and crossbred $V_{\text{E}}$ ($r_{{A_{{v_{pc} }} }}$). Because of the different housing systems, $V_{\text{E}}$ of purebreds consisted of within-individual variance whereas $V_{\text{E}}$ of crossbreds was the sum of within-individual and between-individual variance. Based on simulations with purebred data, we observed that the genetic correlation between $V_{\text{E}}$ of hens in individual cages and $V_{\text{E}}$ of multiple-hen cages was only slightly higher than the $r_{{A_{{v_{pc} }} }}$, which indicated that the difference in definition of $V_{\text{E}}$ had a large contribution to $r_{{A_{{v_{pc} }} }}$. Using the results of the purebred simulations and some algebra, we derived the genetic correlation for $V_{\text{E}}$ between purebreds and crossbreds when the definition of $V_{\text{E}}$ was within-individual variance in both purebreds and crossbreds ($r_{{A_{{v_{w,pc} }} }}$). The difference between $r_{{A_{{v_{w,pc} }} }}$ and $r_{{A_{{v}_{pc} }}}$ indicates the contribution of the difference in definition of $V_{\text{E}}$ to the genetic correlation between purebred and crossbred $V_{\text{E}}$.

We assumed that the within-individual variance was partly determined by its additive genetic effect $A_{{v_{w} }}$ with variance $\sigma_{{A_{{v_{w} }} }}^{2}$. Because in purebreds, $V_{\text{E}}$ was only the within-individual variance, the genetic variance in $V_{\text{E}}$ in purebreds was $\sigma_{{a_{v_{p}} }}^{2} = \sigma_{{A_{{v_{w} }} }}^{2}$. In crossbreds, $V_{\text{E}}$ was the sum of within-individual and between-individual variance, which were both determined by separate additive genetic effects, $A_{{v_{w} }}$ and $A_{{v_{b} }}$, respectively, which could be correlated. Therefore, the genetic variance in $V_{\text{E}}$ for crossbreds was $\sigma_{{A_{{v_{c} }} }}^{2} = \sigma_{{A_{{v_{w} }} }}^{2} + \sigma_{{A_{{v_{b} }} }}^{2} + 2cov_{{A_{{v_{w} }} ,A_{{v_{b} }} }}$. Because of the difference in definition of $V_{\text{E}}$ for purebreds and crossbreds, the $r_{{A_{{v_{pc} }} }}$ was rewritten as:

13 $$\begin{aligned} r_{{A_{{v_{pc} }} }} & = \frac{{cov\left( {A_{{v_{w,p} }} ,A_{{v_{w,c} }} + A_{{v_{b,c} }} } \right)}}{{\sigma_{{A_{{v_{p} }} }} \sigma_{{A_{{v_{c} }} }} }} \\ & = \frac{{r_{{A_{{v_{w,pc} }} }} \sigma_{{A_{{v_{w,p} }} }} \sigma_{{A_{{v_{w,c} }} }} + r_{{A_{{v_{w,p} }} A_{vb,c} }} \sigma_{{A_{{v_{w,p} }} }} \sigma_{{A_{{v_{b,c} }} }} }}{{\sigma_{{A_{{v_{p} }} }} \sigma_{{A_{{v_{c} }} }} }}, \\ \end{aligned}$$

where $\sigma_{{A_{{v_{w,p} }} }}$ is the genetic standard deviation for within-individual variance in purebreds, $\sigma_{{A_{{v_{w,c} }} }}$ is the genetic standard deviation for within-individual variance in crossbreds, $r_{{A_{{v_{w,p} }} A_{{v_{b,c} }} }}$ is the genetic correlation between within-individual variance in purebreds and between-individual variance in crossbreds, $\sigma_{{A_{{v_{b,c} }} }}$ is the genetic standard deviation for between-individual variance in crossbreds, $\sigma_{{A_{{v_{p} }} }}$ is the genetic standard deviation in purebreds for $V_{\text{E}}$, i.e. only within-individual variance ($\sigma_{{A_{{v_{w,p} }} }} = \sigma_{{A_{{v_{p} }} }}$), and $\sigma_{{A_{{v_{c} }} }}$ is the genetic standard deviation for $V_{\text{E}}$ in crossbreds, i.e. the combination of within-individual and between individual variance.

After some rearranging of Eq. 13 and using $\sigma_{{A_{{v_{w,p} }} }} = \sigma_{{A_{{v_{p} }} }}$:

14 $$r_{{A_{{v_{w,pc} }} }} = r_{{A_{v,pc} }} \frac{{\sigma_{{A_{{v_{c} }} }} }}{{\sigma_{{A_{{v_{w,c} }} }} }} - r_{{A_{{v_{w,p} }} A_{{v_{b,c} }} }} \frac{{\sigma_{{A_{{v_{b,c} }} }} }}{{\sigma_{{A_{{v_{w,c} }} }} }}.$$

Equation 14 contained many unknowns, but the simulation with purebred data can provide some of the missing parameters. First of all, we calculated the genetic correlation between $A_{{v_{w} }}$ and $A_{{v_{b} }}$ for purebreds as a proxy for $r_{{A_{{v_{w,p} }} A_{{v_{b,c} }} }}$:

15 $$r_{{A_{{v_{w} ,}} A_{{v_{b} }} }} = \frac{{r_{{A_{{v_{ic} ,}} A_{{v_{mc} }} }} \sigma_{{A_{{v_{mc} }} }} - \sigma_{{A_{{v_{w} }} }} }}{{\sigma_{{A_{{v_{b} }} }} }},$$

where $r_{{A_{{v_{ic} ,}} A_{{v_{mc} }} }}$ is the genetic correlation between $V_{\text{E}}$ of individual cages (IC) and $V_{\text{E}}$ of multiple-hen cages (MC). Furthermore, we estimated $\sigma_{{A_{{v_{b} }} }}^{2}$ in purebreds as:

16 $$\sigma_{{A_{{v_{b} }} }}^{2} = \sigma_{{A_{{v_{mc} }} }}^{2} + \sigma_{{A_{{v_{ic} }} }}^{2} - 2r_{{A_{{v_{ic} ,}} A_{{v_{mc} }} }} \sigma_{{A_{{v_{ic} }} }} \sigma_{{A_{{v_{mc} }} }} ,$$

where $\sigma_{{A_{{v_{mc} }} }}^{2}$ is the estimated genetic variance in $V_{\text{E}}$ of multiple-hen cages in the purebred simulation and $\sigma_{{A_{{v_{ic} }} }}^{2}$ is the estimated genetic variance in $V_{\text{E}}$ of individual cages ($\sigma_{{A_{{v_{ic} }} }}^{2} = \sigma_{{A_{{v_{p} }} }}^{2}$). When applying Eq. 15, the $r_{{A_{{v_{w} ,}} A_{{v_{b} }} }}$ was almost zero in the purebred simulation. Assuming $r_{{A_{{v_{w} ,}} A_{{v_{b} }} }} = 0$, Eq. 14 was simplified to:

17 $$r_{{A_{{v_{w,pc} }} }} = r_{{A_{v,pc} }} \frac{{\sigma_{{A_{{v_{c} }} }} }}{{\sigma_{{A_{{v_{w,c} }} }} }}.$$

Assuming that the proportion of $\sigma_{{A_{{v_{w,c} }} }}^{2}$ and $\sigma_{{A_{{v_{b,c} }} }}^{2}$ to the total genetic variance in $V_{\text{E}}$ of crossbred laying hens $\sigma_{{A_{{v_{c} }} }}^{2}$ was the same in purebreds and crossbreds, we obtained estimates for $\sigma_{{A_{{v_{w,c} }} }}$ and $r_{{A_{{v_{w,pc} }} }}$. To show the effect of $\frac{{\sigma_{{A_{{v_{w,c} }} }} }}{{\sigma_{{A_{{v_{c} }} }} }}$ on $r_{{A_{{v_{pc} }} }}$, Eq. 17 was rearranged to:

18 $$r_{{A_{v,pc} }} = r_{{A_{{v_{w,pc} }} }} \frac{{\sigma_{{A_{{v_{w,c} }} }} }}{{\sigma_{{A_{{v_{c} }} }} }}.$$

Equation 18 shows that $r_{{A_{{v_{pc} }} }}$ decreases when $\frac{{\sigma_{{A_{{v_{w,c} }} }} }}{{\sigma_{{A_{{v_{c} }} }} }}$ decreases, while $r_{{A_{v,pc} }} = r_{{A_{{v_{w,pc} }} }}$ if $\sigma_{{A_{{v_{w,c} }} }} = \sigma_{{A_{{v_{c} }} }}$,which occurs when genetic variation in between-individual variance is absent. In summary, there would be no effect of different definitions of $V_{\text{E}}$ on $r_{{A_{{v_{pc} }} }}$, when genetic variation in the between-individual component of $V_{\text{E}}$ is absent. However, if genetic variation in the between-individual component of $V_{\text{E}}$ exists, the genetic correlation between purebreds and crossbreds is affected not only by the genetic correlation between within-individual variance in purebreds and crossbreds, but also by the proportion of genetic variance in within-individual variance and between-individual variance.

## Additional file

10.1186/s12711-016-0212-2 Format: se_h2v_GCV.f90; Fortran script than can be edited in Notepad, Wordpad, Context or many other editors. Fortran script for calculating standard errors of *h*_*v*_^2^ and *GCV*_*Ve*_.

## References

[1] Hennessy DA (2005). Slaughterhouse rules: animal uniformity and regulating for food safety in meat packing. Am J Agric Econ.

[2] Wolc A, Arango J, Jankowski T, Dunn I, Settar P, Fulton JE (2014). Genome-wide association study for egg production and quality in layer chickens. J Anim Breed Genet.

[3] Zhang LC, Ning ZH, Xu GY, Hou ZC, Yang N (2005). Heritabilities and genetic and phenotypic correlations of egg quality traits in brown-egg dwarf layers. Poult Sci.

[4] Hill WG, Mulder HA (2010). Genetic analysis of environmental variation. Genet Res.

[5] Sell-Kubiak E, Bijma P, Knol EF, Mulder HA (2015). Comparison of methods to study uniformity of traits: application to birth weight in in pigs. J Anim Sci.

[6] Kapell DNRG, Ashworth CJ, Knap PW, Roehe R (2011). Genetic parameters for piglet survival, litter size and birth weight or its variation within litter in sire and dam lines using Bayesian analysis. Livest Sci.

[7] Wolc A, Arango J, Settar P, Fulton JE, O’Sullivan NP, Preisinger R (2012). Genome-wide association analysis and genetic architecture of egg weight and egg uniformity in layer chickens. Anim Genet.

[8] Wei M, van der Werf JHJ (1995). Genetic correlation and heritabilities for purebred and crossbred performance in poultry egg production traits. J Anim Sci.

[9] Besbes B, Gibson JP (1999). Genetic variation of egg production traits in purebred and crossbred laying hens. Anim Sci.

[10] Hidalgo AM, Bastiaansen JWM, Lopes MS, Harlizius B, Groenen MAM, de Koning DJ (2015). Accuracy of predicted genomic breeding values in purebred and crossbred Pigs. G3 (Bethesda).

[11] Habier D, Götz KU, Dempfle L (2007). Estimation of genetic parameters on test stations using purebred and crossbred progeny of sires of the Bavarian Pietrain. Livest Sci.

[12] Serenius T, Stalder KJ, Puonti M (2006). Impact of dominance effects on sow longevity. J Anim Breed Genet.

[13] Wei M, van der Steen HAM (1991). Comparison of reciprocal recurrent selection with pure-line selection systems in animal breeding (a review). Anim Breed Abstr.

[14] Bijma P, Bastiaansen JWM (2014). Standard error of the genetic correlation: how much data do we need to estimate a purebred–crossbred genetic correlation?. Genet Sel Evol.

[15] Cavero D, Schmutz M, Icken W, Preisinger R (2012). Attractive eggshell color as a breeding goal. Lohmann Inf.

[16] Rönnegård L, Felleki M, Fikse F, Mulder HA, Strandberg E (2010). Genetic heterogeneity of residual variance: estimation of variance components using double hierarchical generalized linear models. Genet Sel Evol.

[17] Felleki M, Lee D, Lee Y, Gilmour AR, Rönnegård L (2012). Estimation of breeding values for mean and dispersion, their variance and correlation using double hierarchical generalized linear models. Genet Res (Camb).

[18] Mulder HA, Rönnegård L, Fikse WF, Veerkamp RF, Strandberg E (2013). Estimation of genetic variance for macro- and micro-environmental sensitivity using double hierarchical generalized linear models. Genet Sel Evol.

[19] Hoaglin DC, Welsh RE (1978). The hat matrix in regression and ANOVA. Am Stat.

[20] Mulder HA, Bijma P, Hill WG (2007). Prediction of breeding values and selection responses with genetic heterogeneity of environmental variance. Genetics.

[21] Sae-Lim P, Kause A, Janhunen M, Vehvilainen H, Koskinen H, Gjerde B (2015). Genetic (co)variance of rainbow trout (*Oncorhynchus mykiss*) body weight and its uniformity across production environments. Genet Sel Evol.

[22] Felleki M, Lundeheim N (2012). Genetic control of residual variance in teat number in pigs. Proc Assoc Adv Anim Breed Genet.

[23] Lynch M, Walsh B (1998). Genetics and analysis of quantitative traits.

[24] Sell-Kubiak E, Duijvesteijn N, Lopes MS, Janss LLG, Knol EF, Bijma P (2015). Genome-wide association study reveals novel loci for litter size and its variability in a Large White pig population. BMC Genom.

[25] Lee Y, Nelder JA (2006). Double hierarchical generalized linear models. Appl Stat.

[26] Wolc A, Arango J, Settar P, Fulton JE, O’Sullivan NP, Preisinger R (2013). Analysis of egg production in layer chickens using a random regression model with genomic relationships. Poult Sci.

[27] Hansen TF, Pelabon C, Houle D (2011). Heritability is not evolvability. Evol Biol.

[28] Houle D (1992). Comparing evolvability and variability of quantitative traits. Genetics.

[29] Schaeffer LR, Dekkers JCM. Random regressions in animal models for test-day production in dairy cattle. In Proceedings of the 5th world congress on genetics applied to livestock production, 7–12 Aug 1994; Guelph. 1994;18:443–6.

[30] Meyer K, Kirkpatrick M (2005). Up hill, down dale: quantitative genetics of curvaceous traits. Philos Trans R Soc B Biol Sci..

[31] Ibanez-Escriche N, Varona L, Sorensen D, Noguera JL (2008). A study of heterogeneity of environmental variance for slaughter weight in pigs. Animal.

[32] Bijma P, Van Arendonk JAM (1998). Maximising genetic gain for the sire line of a crossbreeding scheme utilising both purebred and crossbred information. Anim Sci.

[33] Mulder HA, Bijma P, Hill WG (2008). Selection for uniformity in livestock by exploiting genetic heterogeneity of residual variance. Genet Sel Evol.

